# Radiomic Feature-Based Predictive Model for Microvascular Invasion in Patients With Hepatocellular Carcinoma

**DOI:** 10.3389/fonc.2020.574228

**Published:** 2020-11-05

**Authors:** Mu He, Peng Zhang, Xiao Ma, Baochun He, Chihua Fang, Fucang Jia

**Affiliations:** ^1^ The First Department of Hepatobiliary Surgery, Zhujiang Hospital, Southern Medical University, Guangdong Provincial Clinical and Engineering Center of Digital Medicine, Guangzhou, China; ^2^ Research Laboratory for Medical Imaging and Digital Surgery, Shenzhen Institutes of Advanced Technology, Chinese Academy of Sciences, Shenzhen, China

**Keywords:** hepatocellular carcinoma, microvascular invasion, radiomics, nomogram, computed tomography

## Abstract

**Objective:**

This study aimed to build and evaluate a radiomics feature-based model for the preoperative prediction of microvascular invasion (MVI) in patients with hepatocellular carcinoma.

**Methods:**

A total of 145 patients were retrospectively included in the study pool, and the patients were divided randomly into two independent cohorts with a ratio of 7:3 (training cohort: n = 101, validation cohort: n = 44). For a pilot study of this predictive model another 18 patients were recruited into this study. A total of 1,231 computed tomography (CT) image features of the liver parenchyma without tumors were extracted from portal-phase CT images. A least absolute shrinkage and selection operator (LASSO) logistic regression was applied to build a radiomics score (Rad-score) model. Afterwards, a nomogram, including Rad-score as well as other clinicopathological risk factors, was established with a multivariate logistic regression model. The discrimination efficacy, calibration efficacy, and clinical utility value of the nomogram were evaluated.

**Results:**

The Rad-score scoring model could predict MVI with the area under the curve (AUC) of 0.637 (95% CI, 0.516–0.758) in the training cohort as well as of 0.583 (95% CI, 0.395–0.770) in the validation cohort; however, the aforementioned discriminative approach could not completely outperform those existing predictors (alpha fetoprotein, neutrophilic granulocyte, and preoperative hemoglobin). The individual predictive nomogram which included the Rad-score, alpha fetoprotein, neutrophilic granulocyte, and preoperative hemoglobin showed a better discrimination efficacy with AUC of 0.865 (95% CI, 0.786–0.944), which was higher than the conventional methods’ AUCs (nomogram vs Rad-score, alpha fetoprotein, neutrophilic granulocyte, and preoperative hemoglobin at P < 0.001, P = 0.025, P < 0.001, and P = 0.001, respectively). When applied to the validation cohort, the nomogram discrimination efficacy was still outbalanced those above mentioned three remaining methods (AUC: 0.705; 95% CI, 0.537–0.874). The calibration curves of this proposed method showed a satisfying consistency in both cohorts. A prospective pilot analysis showed that the nomogram could predict MVI with an AUC of 0.844 (95% CI, 0.628–1.000).

**Conclusions:**

The radiomics feature-based predictive model improved the preoperative prediction of MVI in HCC patients significantly. It could be a potentially valuable clinical utility.

## Introduction

Hepatocellular carcinoma (HCC) is considered as the sixth most high incidence cancer and HCC is also as the world’s third leading cause of cancer deaths. Among the primary cancer of the liver, HCC is the predominant pathological type and has became a significant public health-care concern (1, 2). Among the current treatment strategies for HCC, the optimal and most efficient treatment is radical surgical resection. In the past several decades, along with the development of modern medical imaging technology and improved surgical skills, the success ratio of hepatectomy for HCC has increased continuously (3). In spite of this, the postoperative five-year recurrence ratio of HCC is approximately 70%, which is unsatisfactory and indicates a significant influence on overall survival for HCC patients (4). Microvascular invasion (MVI) is a crucial predictive factor for recurrence among HCC patients who underwent hepatoectomy for tumor resection or received liver transplant surgery, and most patients with early recurrence were pathologically verified as MVI positive (5, 6). Therefore, prediction for MVI before surgery has significant clinical value for decision making, postoperative adjuvant therapy and comprehensive prognostic evaluation of patients with HCC.

Tumor macrovascular invasion of hepatic vein and/or portal vein in HCC patients can be identified through contrast-enhanced computed tomography (CECT) or magnetic resonance imaging (MRI) examination before surgery (7). Conversely, microvascular invasion (MVI) cannot be detected directly by means of preoperative radiologic examination. In addition, the effectiveness of preoperative pathological biopsy for MVI is unsatisfactory to some extent. Hence, the gold standard to verify the occurrence of MVI is to evaluate the surgical specimen slices through preoperative pathological examination (i.e., H&E staining, etc.) which limits its application as a predictive factor for comprehensive prognosis assessment (8). Imaging markers are emerging as preoperative predictive factors for tumor heterogeneity that have the potential to predict MVI in a high-throughput, high-precision, and noninvasive manner. Current published studies have indicated that the correlation between the MVI and imaging features could be quantitively analyzed for further research on the occurrence and progression of MVI. Imaging features could be extracted from the following medical imaging modalities: contrast-enhanced CT, gadolinium-ethoxybenzyl-diethylenetriamine penta-acetic acid (Gd-EOB-DTPA; EOB Primovist^®^)-enhanced magnetic resonance imaging (MRI) and ^18^F-fluorodeoxyglucose positron emission tomography (PET). In addition, those imaging features incorporated laborator testing, quantitative or qualitative results to establish predictive models to classify whether patients with HCC are MVI positive or negative, which is of great value for surgeons to evaluate liver transplant recipients or HCC therapy scheme decision making (9–11). In addition, radiomics is an emerging methodology for medical imaging analysis that applies machine learning algorithms and statistical analysis software to obtain high-throughput radiological features for prognosis prediction or tumor-related overall survival analysis. In the past, our research group’s study results indicated that radiomics-based predictive models have the potential to predict the occurrence of posthepatectomy liver failure and the occurrence of postpancreaticoduodenectomy pancreatic fistula among a study cohort with satisfactory predictive performance (12, 13). Recent studies showing predictive radiomics models based on contrast-enhanced CT (CECT) as an easy-to-use, efficient, and noninvasive prediction method for MVI have provided reliable instruction for the definite diagnosis of whether HCC patients had MVI preoperatively (14, 15). Precise prediction of MVI before surgery has significant clinical implications for issues such as efficient stratification of treatment strategies, optimization of treatment schemes, and establishment of comprehensive surgery planning for HCC patients with MVI positivity or negativity. Due to the specific biological characteristics of MVI, the contrast agent in CECT likely enters the vessels of tumors and then diffuses into the microvessels over the range of one centimeter around the primary lesion. An enlarged surgical margin (usually over one centimeter) was verified to decrease the postoperative tumor recurrence ratio, indicating the presence of MVI, among patients with HCC (16, 17). For patients who have a tumor diameter less than three centimeters and are highly suspected to be MVI positive, anatomical hepatectomy is necessary. For non-anatomical hepatectomy, the surgical margin should be over one centimeter from the primary lesion (18, 19). Compared with surgical resection, radiofrequency ablation is not a rational option for small (diameter under three centimeters) HCC patients combined with MVI because of high postoperative recurrence ratio (20).

The radiomic predictive model incorporates clinical predicative factors and imaging characteristic features into a reliable and efficient MVI risk-stratification scoring system, which helps clinical practitioners provide optimal treatment schemes for HCC patients.

In the present study, we aimed at build as well as evaluate a radiomics nomogram based on CT imaging textural features incorporated with clinically related risk factors for MVI occurrence prediction among HCC patients before surgery.

## Materials and Methods

### Patients

The data originated from 146 patients who received liver resection at Zhujiang Hospital, Southern Medical University, between January 2012 and December 2018, were retrospectively collected and analyzed. The patients with HCC were carefully selected, and the inclusion standards as indicated below: (1) Be at least 18 years old, both male and female were included; (2) contrast-enhanced CT scans were performed within one week before surgery; and (3) all surgical specimens were confirmed to be HCC by MVI evaluation and histopathological examination. The exclusion standards were as follows: (1) previous hepatectomy; (2) HCC lesion boundaries not clearly visible on CT images; (3) preoperative treatment, including transhepatic arterial chemotherapy and embolization, portal vein embolization, radiofrequency ablation therapy, targeted cancer therapy, etc.; and (4) incomplete clinical data or pathology data. Patients were randomly allocated into two independent cohorts as training cohort and validation cohort according to a ratio of 7:3. An additional 18 patients between January and December 2019 were enrolled in a prospective pilot analysis, and all patients signed the informed consent. The Ethics Review Board of Zhujiang Hospital, Southern Medical University, approved this study and supervised the procedures.

### Histopathological Analysis

The final diagnostic results, including MVI grade, were based on pathologic reports of surgical specimens. On the basis of the Clinical Practice Guidelines of Chinese Society of Pathology (21), the definite diagnosis of MVI was based on the pathological standard as follows: MVI was graded according to the number of cells seen in the endothelial vascular lumen by microscopic examination, and then MVI was categorized as three additional subgrades, M0 indicating no MVI; M1 for fewer than five MVI occurrence in adjoining liver parenchyma and the distance from tumors was under one centimeter (which is also regarded as the low-risk group); and M2 for more than five MVI in adjoining liver parenchyma and the distance from tumors was greater than one centimeter (regarded as the high-risk group). All specimen slices were reviewed and analyzed by two pathologists who had at least 10 to 15 years of hepatic pathology experience. Aforementioned two specialists were blinded to the related clinicopathological as well medical imaging examination information of the patients who were included in the study cohort. When there was a disagreement, a consistency review was performed.

### CT Data Collection

All CT examinations were finished in our hospital spiral CT (128 -slice, Siemens Medical system, Erlangen, Germany). To ensure the consistency of the image data, all the scanning parameters were set as follows: 120 kV, 250–300 mAs, and a matrix of 512 × 512. The slice thickness and interval were set as one mm and zero mm, respectively, and image reconstruction was set to 1 mm. To obtain contrast-enhanced image data, 90 ml iodine contrast agent (iodine concentration: 350 mg/ml) was administered *via* a vein by a contrast injector at a speed of 3 milliliter per second, and dynamic contrast-enhanced imaging data acquisition was performed at fixed time points: for the arterial phase, acquisition occurred at approximately 30–33 s after administration; for the portal vein phase, it was 67–70 s, and for the equilibrium phase, 177–180 s. After the entire acquisition procedure was completed, we selected the portal venous phase raw data for texture analysis. All the medical images in this study were collected from the Pictures Archiving and Communication System at our hospital radiology department.

### Radiomics Feature Extraction

**Figure 1:**
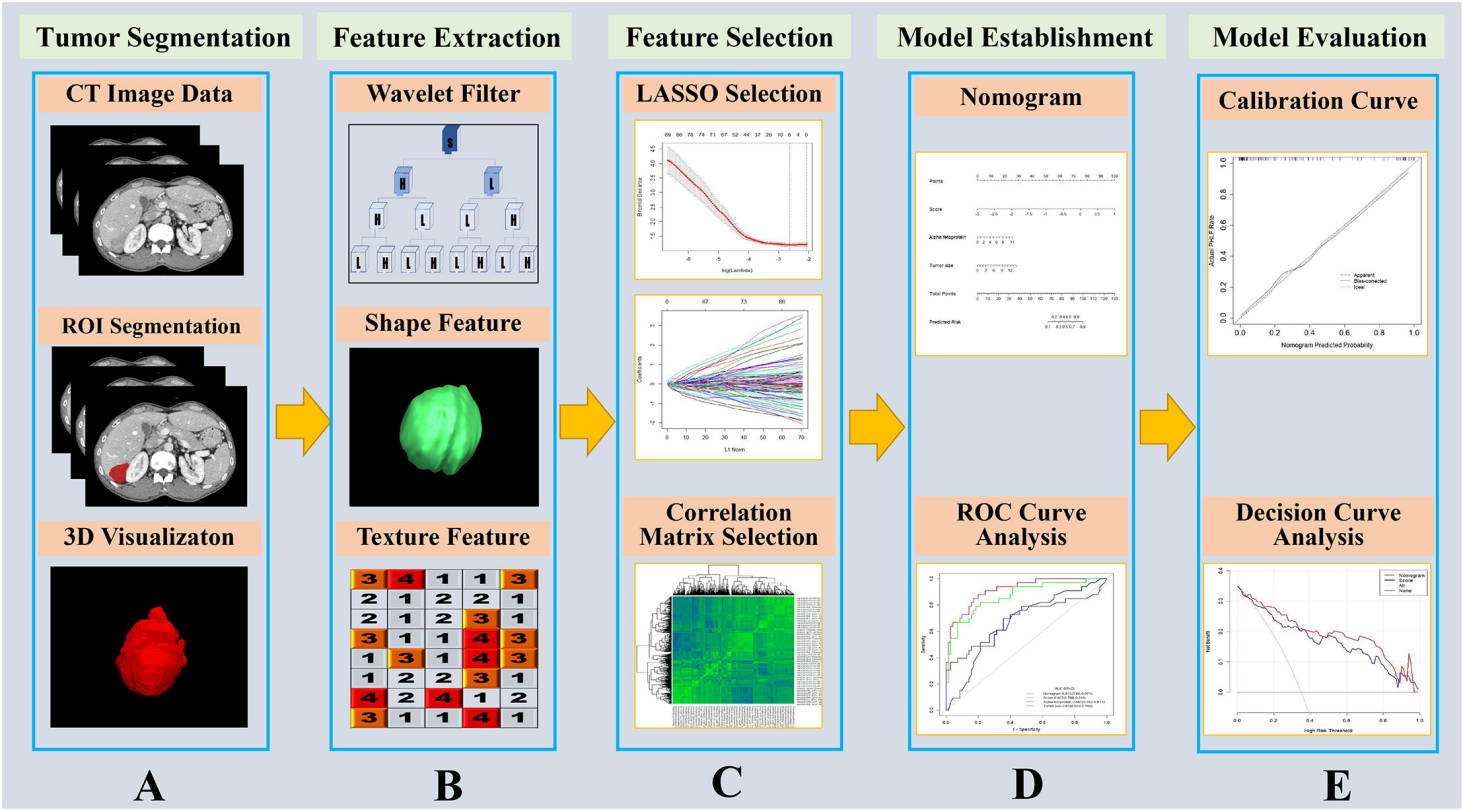
Flow chart of the study. **(A)** The portal vein contrast-enhanced computed tomography (CECT) image was used as input, and the liver was segmented by homemade segmentation software (22) and manually corrected. The tumor ROI was contoured by a radiologist with more than 10 years of experience. **(B)** The liver parenchyma without tumor was set as the ROI image, and features containing as shape related features, texture related features and wavelet filtered features which were extracted by PyRadiomics software (23). **(C)** The extracted features were reduced *via* least absolute shrinkage and selection operator (LASSO) and correlation matrix selection. **(D)** The radiomics features and other clinicopathological variables were applied to establish a nomogram for predict microvascular invasion, and ROI curve analysis was conducted. **(E)** The nomogram model was evaluated with calibration curve and decision curve analysis.

The holistic radiomics modeling procedure was shown in **Figure 1**. We extracted a total of 1,231 CT image features from the liver parenchyma without tumors. The extracted image features were categorized into four types as follows: diagnostic intensity for liver parenchyma (N=13), original hepatic textural feature (N=100), tumor size and shape-based feature (N=430), and wavelet (N=688). The mathematical descriptions of each corresponding feature above can be found in the website of the open-source application PyRadiomics (https://pyradiomics.readthedocs.io/en/latest/).

### Data Analysis

One hundred forty-five patients were included in the study pool, therefore, those patients were randomly allocated into two independent cohorts according to a ratio of 7:3 (training cohort: n = 101, validation cohort: n = 44). An additional 18 patients were also enrolled into this study for a prospective pilot analysis which was aimed to evaluate its underlying clinical practice potency.

A histologic diagnosis of tumor with microvascular invasion or tumor without microvascular invasion was entered as a dependent variable in a least absolute shrinkage and selection operator (LASSO) logistic regression algorithm. As to the LASSO logistic regression, we applied 10-fold cross validation method to identified the optimal regularization parameter lambda, which reduces the mean cross-validation error at maximum extent. those image features who were considered as non-zero coefficients within the LASSO regression model were regarded as most predictive radiomics features and were chosen for calculating the radiomics score. The radiomics score was calculated through a formula which in brief was a semplice linear combination of the selected radiomics based features multiplied by their corresponding coefficients.

The radiomics score as well as other clinicopathological risk variables, including age, sex, tumor sizes, viral hepatitis etiology, drinking history, alpha fetoprotein content, tumor numbers, neutrophil counts, lymphocyte counts, neutrophil-lymphocyte ratio, preoperative alanine transaminase, preoperative aspartate aminotransferase, preoperative total bilirubin, preoperative hemoglobin, preoperative albumin, preoperative blood platelet counts, preoperative prothrombin time, and histopathological differentiation degree, were used in a multivariate logistic regression model as predictive factors for tumors with microvascular invasion or tumors without microvascular invasion among training cohort. The optimal model selection was performed applied a backward stepwise selection procedure through utilizing likelihood ratio testing with Akaike information criterion (AIC). Nomogram was constructed based on those significant features in the multivariate logistic regression model among which radiomics score as well as clinicopathological risk factors were included.

The nomogram’s predictive accuracy was evaluated by three methods. First, discrimination efficacy was assessed through the result of the area under the area under curve (AUC) with values ranging from 0.5 (which means mentioned model possess no discrimination potent) to 1 (which means mentioned model possess a perfect discrimination potent). Second, by plotting the predicted probability of the nomogram relative to the ratio of the observed events on a series of equidistant values within the range of predicted probabilities, the power of calibration can be evaluated visually. In addition, decision curve analysis (DCA) was carried out to tell physicians the interval of threshold probabilities to judge whether the prediction model would be of clinical benefit or not. If the typical threshold probabilities of tumors with microvascular invasion or tumors without microvascular invasion at which surgeons would opt for comprehensive treatment lie within a certain benefit range, the model is of clinical value.

### Statistical Analysis

For statistical analysis in this study an open source software R (version 3.6.1) was applied. (https://www.r-project.org). consider the various type of data, we employed different statistical test as below: for continuous variables, the Mann-Whitney U test were used for the comparison. Correspondingly, as for categorical variables, the Chi-square test was used for the comparison. in order to conduct LASSO logistic regression analysis we used “glmnet” package, “pROC” package was applied for calculation and comparisons of AUC and ROC plotting of each model as well as for nomogram training and calibration “rms” package was employed, in addition for decision curve analysis we chosen “rmda” package to conduct it. A two-sided *P* value < 0.05 was considered as indicative of statistical significance. For the final independent predictors included in this prediction model selection, we applied both univariant logistic regression and multivariant logistic regression and the factors whose *P* value < 0.05 were considered as indicative of final independent predictors.H

## Results

### Patient Demographics and Clinicopathological Characteristics

The demographics and clinicopathological characteristics of the study participants are displayed in **Table 1**. It is clearly that there were no differences between the training cohort and the validation cohort. The median ages of these two cohorts were 50.0 and 47.5 years, respectively. As for patient gender in both cohort male was ponderance proportion (81.2% and 84.1%, respectively). In addition, for positive occurrence ratio there were 30 (29.7%) and 16 (36.4%) patients who experienced tumors with microvascular invasion among training cohort as well validation cohort, respectively. For patients among pilot study, those data are displayed in **Table 2**.

**Table 1:** Demographic and clinicopathologic characteristics of the study participants.

Characteristic	Training cohort (N=101)	Validation cohort (N=44)	*P* value
Age, median (IQR), years	50.00 (41.00–58.00)	47.50(43.00–59.50)	0.957
Sex, N (%)			
Female	19 (18.8)	7 (15.9)	0.854
Male	82 (81.2)	37 (84.1)	
Etiology of viral hepatitis, N (%)			
None	22 (21.8)	9 (20.5)	0.291
Hepatitis B	73 (72.3)	31 (70.5)	
Hepatitis C	3 (3.0)	2 (4.5)	
Hepatitis E	2 (2.0)	0 (0.0)	
Hepatitis B + C	0 (0.0)	2 (4.5)	
Hepatitis B + E	1 (1.0)	0 (0.0)	
Drinking history, N (%)			
No	87 (86.1)	38 (86.4)	1.000
Yes	14 (13.9)	6 (13.6)	
Alpha fetoprotein content, median (IQR)	3.16 (1.46–6.36)	2.15 (0.82–4.94)	0.067
Number of tumors, median (IQR)	1.00 (1.00–1.00)	1.00 (1.00–1.00)	0.469
Neutrophil counts, median (IQR)	3.84 (3.19–4.90)	3.93 (2.96–4.95)	0.877
Lymphocyte counts, median (IQR)	1.74 (1.30–2.20)	1.69 (1.33–2.15)	0.877
Neutrophil-lymphocyte ratio, median (IQR)	2.26 (1.65–2.97)	2.41 (1.62–3.33)	0.752
Preoperative alanine transaminase, median (IQR)	35.00 (20.00–51.00)	31.50(20.50–47.25)	0.380
Preoperative aspartate aminotransferase, median (IQR)	35.00 (23.00–45.00)	31.00(23.50–44.00)	0.523
Preoperative total bilirubin, median (IQR)	11.50 (8.00–16.40)	10.30 (8.75–14.60)	0.940
Preoperative hemoglobin, median (IQR)	135.00(120.00–149.00)	134.50(120.75–151.00)	0.908
Preoperative albumin, median (IQR)	39.00 (36.00–41.60)	40.70(35.95–42.73)	0.541
Preoperative blood platelet counts, median (IQR)	172.00(143.00-246.00)	195.00(141.25–256.00)	0.563
Preoperative prothrombin time, median (IQR)	13.70 (13.00–14.20)	13.45(12.67–14.20)	0.242
Histopathological differentiation degree, N (%)			
Poor	64 (63.4)	27 (61.4)	0.438
Moderate	22 (21.8)	7 (15.9)	
Well	15 (14.9)	10 (22.7)	
Tumor with or without microvascular invasion (%)			
With	71 (70.3)	28 (63.6)	0.550
Without	30 (29.7)	16 (36.4)	

**Table 2:** Demographic and clinicopathologic characteristics of the pilot participants.

Characteristic	Pilot (N=18)
Age, median (IQR), years	52.00 (48.25–57.25)
Sex, N (%)	
Female	3 (16.7)
Male	15 (83.3)
Etiology of viral hepatitis, N (%)	
Hepatitis B	10 (55.6)
None	8 (44.4)
Drinking history, N (%)	
No	18 (100.0)
Alpha fetoprotein content, median (IQR)	7.70 (4.42–23.05)
Number of tumors, median (IQR)	1.00 (1.00–1.00)
Neutrophil counts, median (IQR)	3.54 (3.07–4.67)
Lymphocyte counts, median (IQR)	2.02 (1.51–2.68)
Neutrophil-lymphocyte ratio, median (IQR)	1.89 (1.15–2.38)
Preoperative alanine transaminase, median (IQR)	32.00 (21.00–57.75)
Preoperative aspartate aminotransferase, median (IQR)	28.00 (24.25–39.25)
Preoperative total bilirubin, median (IQR)	14.25 (10.92–15.20)
Preoperative hemoglobin, median (IQR)	143.50 (124.50–148.25)
Preoperative albumin, median (IQR)	36.80 (34.55–40.28)
Preoperative blood platelet counts, median (IQR)	195.00 (172.50–250.75)
Preoperative prothrombin time, median (IQR)	13.35 (12.70–13.85)
Histopathological differentiation degree, N (%)	
Poor	13 (72.2)
Moderate	1 (5.6)
Well	4 (22.2)
Tumor with or without microvascular invasion, N (%)	
With	11 (61.1)
Without	7 (38.9)

### Development of the Rad-Score

**Figure 2:**
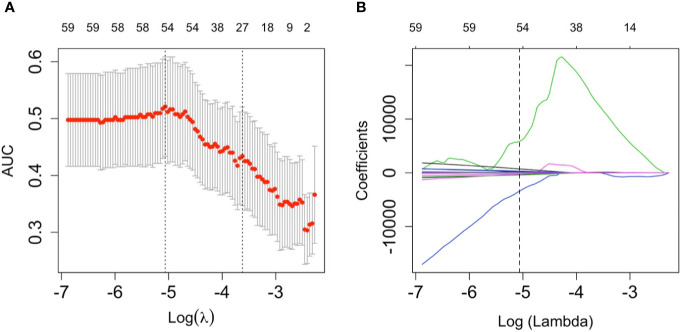
The least absolute shrinkage and selection operator (LASSO) logistic regression algorithm was employed to determine the most significant radiomics features in terms of microvascular invasion (MVI) prediction as well calculated for the Rad-score. **(A)** a ten-fold cross validation method was employed to select the optimal lambda parameter contained in the LASSO algorithm that minimized the mean squared error. The mean-squared errors between upper bound and lower bound standard deviations were plotted according to the lambda changing sequence. The vertical dotted lines in the graph stands for the two selected lambdas. The lambda on the left corner of graph provided the minimum mean cross-validation error, meanwhile, the lambda on the right corner of graph provided the most optimal regularized model which the error within one minimal standard error, therefore, the optimal lambda value 0.0063, whose log(lambda)= -5.0637, was employed to feature selection. **(B)** A coefficient profile plot of the LASSO model was produced. Every single curve represents the trajectory changing of each independent predictor. The greater lambda is, the more the coefficients are reduced. The vertical dotted line indicates the optimal lambda, which results in two coefficients.

A total of 1,231 features were extracted from the image data for each patient. These 1,231 features were reduced to 2 possible indicators based on the 101 patients among training cohort. The Rad-score formula constructed through a simple linear combination of aforementioned 2 indicators multiplied by LASSO coefficients as follows: Rad-score=55186.42 + 37645.32*log-sigma-3-0-mm-3D_gldm_SmallDependenceLowGrayLevelEmphasis - 46900.48 * log-sigma-5-0-mm-3D_glcm_Idmn (**Figure 2**). Patients who had tumors without MVI obtained relatively lower Rad-scores generally than patients who suffered from tumors combined with microvascular invasion. This discrepancy was detected between on Rad-scores (median (interquartile range)) among training cohort (−2.567 (-6.731~1.592) vs 0.164 (-5.160~4.068), respectively, *P*=0.013) also among the validation cohort (-2.477 (-7.465~0.864) vs 0.123 (-6.434~3.899), respectively, *P*=0.369).

### Development, Validation, and Assessment of the Nomogram

**Figure 3:**
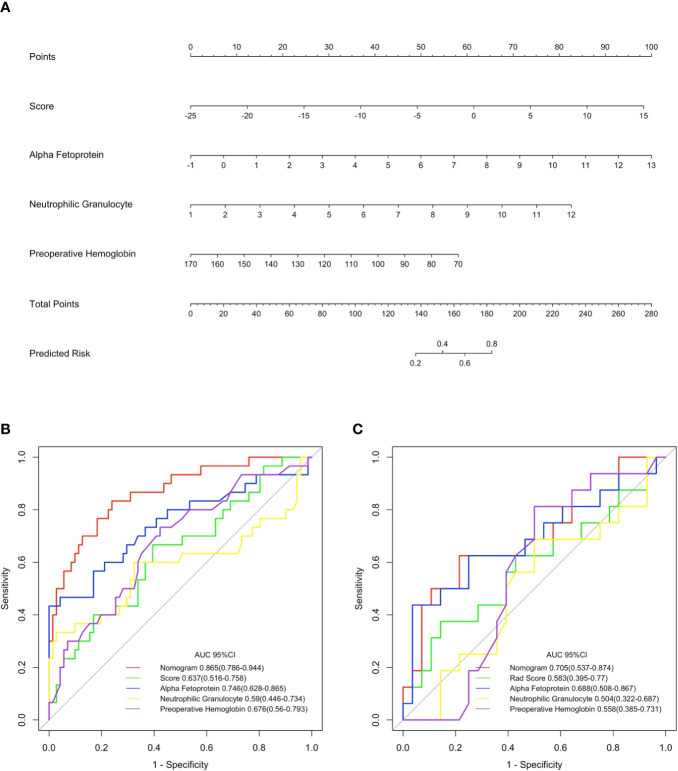
Nomogram for the prediction of the tumor with microvascular invasion occurring or not and its discrimination performance. **(A)** data among the training cohort were employed to establish the purposed monogram which the Rad-score, alpha fetoprotein, neutrophilic granulocyte and preoperative hemoglobin were incorporated. Instructions for reading the nomogram: Find the Rad-score in the Rad-score axis; a straight line was drew to the point axis and the intersection point stands for actual points of the predicted probability which a patient with microvascular invasion (MVI) occurrence or not according to the own Rad-score; repeat above mentioned procedures for another predictors each straight line was drew to the points axis; calculated the total points obtained from each predictor and located it on the total points axis; draw a line perpendicular to the predict risk axis to determine the patient’s risk (24). **(B, C)** display the result of ROC curves analysis for the nomogram and each individual predictor in predicting whether tumors with microvascular invasion occurred among the training and validation cohorts, respectively.

**Figure 4:**
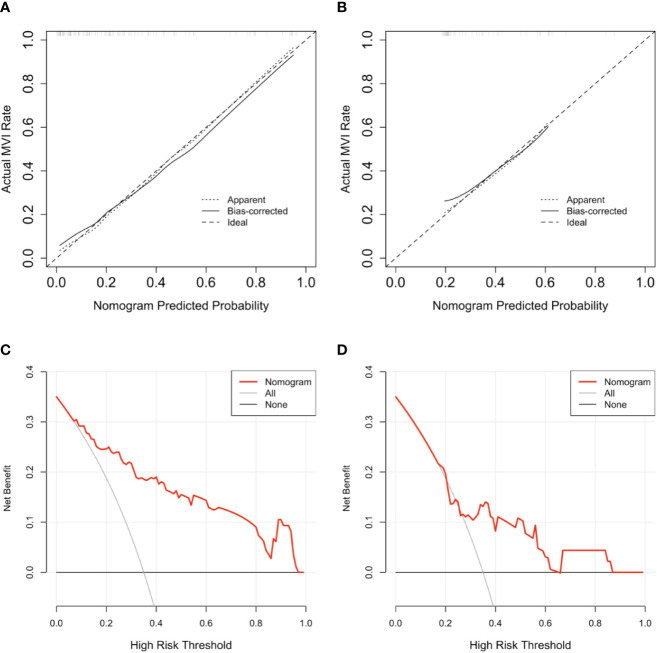
The result of calibration curve analysis as well decision curve analysis for the purposed nomogram. **(A, B)** show the calibration curves analysis for the nomogram in predicting whether tumors with microvascular invasion occurrence among training cohort as well as validation cohort, respectively. the dashed line stands for perfect prediction, the dotted line represents apparent estimates of predicted vs. observed values, meanwhile the solid line on behalf of bias correcting estimates employing 1,000 bootstrap sampling. **(C, D)** show the result of decision curve analysis for the nomogram in predicting whether tumors with microvascular invasion occurrence among training cohort as well as validation cohort, respectively. The result of decision curve analysis indicated that assume the threshold probability that is located in an interval of 0.2 to 0.86, microvascular invasion (MVI) prediction using the nomogram can give more net benefit than by treating either no or all patients in all cohorts.

**Figure 5:**
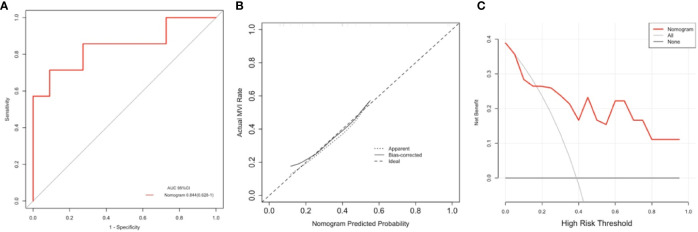
The area under the curve (AUC) **(A)**, calibration curve **(B),** and decision curve analysis (DCA) **(C)** of the nomogram to prospectively analyze the pilot cohort. This purposed radiomics feature-based nomogram was able to predict tumors combined microvascular invasion (MVI) occurrence with an AUC of 0.844 (95% confidence interval:0.628–1.000) **(A)** the result calibration curve showed that the radiomics nomogram was with the ideal line **(B)** and as to result of the DCA represented that at the situation where the threshold probability was within a range from 0.2 to 1.0 **(C)**, this purposed nomogram obtained greater net benefit than either “treat none” or “treat all” scheme, suggesting that this purposed nomogram is still a good clinical utility.

Both univariate and multivariate logistic regression analysis were conducted combining the Rad-score as well as clinical stage among training cohort. The backward stepwise selection process selected Rad-score, alpha fetoprotein content, neutrophil counts, and preoperative hemoglobin as the final independent predictors. A prediction model was constructed and is shown as a nomogram in **Figure 3**. The odds ratios of the prediction model are presented in **Table 3**. Among the training cohort, the AUC of the nomogram was 0.865 (95% CI 0.786–0.944). among validation cohort, the AUC was 0.705 (95% CI: 0.537–0.874). The AUC value of the ROC curve of the nomogram was significantly higher than that of each independent predictor (nomogram vs Rad-score, alpha fetoprotein, neutrophilic granulocyte, and preoperative hemoglobin at P < 0.001, P = 0.025, P < 0.001, and P = 0.001, respectively). The particular results of the discriminatory efficiency are shown in **Figures 3C, D**). This indicates that the Rad-score incorporated with another three clinicopathological factors possessed a greater diagnostic efficiency than any single independent predictors. The calibration curve analysis of this purposed predictive model presents good agreement among our research for either the training cohort or validation cohort (**Figures 4** and **5**).

**Table 3:** Risk factors for the diagnosis of tumors with microvascular invasion.

Characteristic	Univariate logistic regression		Multivariate logistic regression	
	OR (95% CI)	*P* value	OR (95% CI)	*P* value
Score	1.093(1.017-1.176)	0.016*	1.158(1.041-1.29)	0.007*
Age	0.991(0.958-1.025)	0.596	NA	NA
Sex	0.897(0.305-2.635)	0.843	NA	NA
Size of tumors	0.974(0.849-1.117)	0.705	NA	NA
Etiology of viral hepatitis	1.077(0.371-3.128)	0.892	NA	NA
Drinking history	1.969(0.618-6.269)	0.252	NA	NA
Alpha fetoprotein content	1.447(1.216-1.721)	<0.001*	1.533(1.22-1.927)	<0.001*
Number of tumors	0.623(0.111-3.507)	0.592	NA	NA
Neutrophil counts	1.379(1.09-1.745)	0.007*	1.567(1.138-2.158)	0.006*
Lymphocyte counts	0.849(0.441-1.634)	0.624	NA	NA
Neutrophil-lymphocyte ratio	1.212(0.956-1.536)	0.113	NA	NA
Preoperative alanine transaminase	1.004(0.995-1.013)	0.416	NA	NA
Preoperative aspartate aminotransferase	1.007(0.999-1.015)	0.081	NA	NA
Preoperative total bilirubin	0.995(0.967-1.024)	0.747	NA	NA
Preoperative hemoglobin	0.966(0.944-0.989)	0.004*	0.966(0.938-0.995)	0.022*
Preoperative albumin	0.961(0.883-1.046)	0.355	NA	NA
Preoperative blood platelet counts	1.007(1.003-1.012)	0.003*	NA	NA
Preoperative prothrombin time	1.035(0.75-1.427)	0.835	NA	NA
Histopathological differentiation degree	2.765(1.014-7.538)	0.047*	NA	NA

*P < 0.05.

The DCA curve showed that as threshold probability was within a range from 0.1 to 1.0, this nomogram could obtain a greater net benefit than either “treat none” or “treat all” scheme. The nomogram also received higher net benefit than the Rad-score alone across the reasonable threshold probabilities range.

## Discussion

In this study, we established as well assessed radiomics model based on CT imaging textural features and clinically related risk factors for accurate prediction of MVI of HCC before surgical resection. The efficacy of this predictive model was average-to-good but did not totally outperform other current predictive models or scoring systems. Therefore, we developed and evaluated a predictive nomogram based on radiological characteristics to predict the incidence of MVI. Our predictive nomogram included factors such as AFP, NLR, routine blood tests, liver function, and pathological examination results and a combination of potential MVI predictive imaging features. Our results were consistent with previous studies; higher serum AFP level, NLR or poorly differential tumor cell type had a positive correlation with the incidence of MVI among patients with HCC. Through the results of decision curve analysis, independent predictive risk factors such as AFP, NLR, or radiomics features had beneficial effects on improving the predictive power of MVI, which was consistent with current studies; however, the diagnostic efficacy of the model did not outperform other existing predictive models or scoring systems. When we combined the above independent predictive risk factors into a comprehensive diagnostic nomogram, the diagnostic efficacy exceeded the predictive power of any single predictive factor or a combination of predictive factors, and its performance showed satisfactory consistency in both the training cohort and validation cohort. The aforementioned nomogram could improve the individual predictive accuracy of MVI for patients with HCC preoperatively, and the results of decision curve analysis showed that this practical nomogram had the potential to be applied in daily clinical practice.

The diagnosis of HCC depends on radiological examination, such as CT imaging or MRI, to a large extent, which makes it preferable to perform quantitative or qualitative imaging analysis based on the radiomics method. The radiomics method can extract a large number of specific imaging features from radiological images, such as CT images, magnetic resonance images or PET-CT images, in a high-throughput and automatic manner and then utilize those radiomics imaging features with clinicopathological data to establish statistical models for judgment of tumor characteristics or optimal treatment choice and prognosis for individual patients (25). Therefore, radiomics can connect medical imaging and personalized medicine. However, due to the lack of a comprehensive standardized assessment of clinical significance and scientific integrity in previously radiomics studies, it is difficult to promote the application of radiomics image analysis methods among different institutions (26). In current clinical practice, for the reason that there are no known single and highly reliable predictive factors for the incidence of MVI, combining multiple MVI status-related factors with radiomics signatures to build multivariate radiomic models is a feasible option. Previous published studies have utilized multiple imaging modal images (e.g., CT, MR, ultrasound) with radiomics algorithms to predict the incidence of MVI preoperatively and then achieve the ultimate goal of assisting in choosing an optimal surgical treatment or predicting the early recurrence of HCC to provide a reasonable treatment scheme for those patients (27–29). With the rapid development and improvement of radiomics technology, we are able to identify high risky HCC patients with MVI with higher accuracy and better efficacy before surgery which have great significance in terms such as select appropriate surgical therapy strategy, give assistance to confirm rational resection scope to achieve radical hepatoectomy for HCC patients rather than barely refer to tumor location, size or two-dimensional spatial relationship between intrahepatic vasculatures as conventional method did consider of the high postoperative recurrence ratio, for patients whose tumor size lower than three millimeters also susceptible to MVI positive anatomical hepatoectomy are recommended as first choice than radiofrequency ablation, for patients susceptible to MVI positive when select to perform non-anatomical hepatoectomy, the distance from resection margin tumor should beyond one millimeter to decrease the risk of postoperative recurrence (20). Accurately prediction of MVI occurrence has contributed to achieve radical tumor resection for patients with HCC in addition prolong the disease-free survival to the maximum extent. MVI as a significant factor which influence long term survival rate, radiomics features based prediction model applied to this crucial issue will provide more effective guidance for subsequent clinical researches and therapy scheme making.

Related studies have shown that approximately 85% of MVI is located within one millimeter (which is called the peritumoral area) of the margin of tumors (30). In this research, we extracted both peritumoral and intratumoral CT radiomics features to establish a nomogram model. Zhao et al. (31) established a scoring system using predictive factors such as intratumoral arteries, nonnodular HCC type also the absence of a tumor vessel based on CECT images to predict MVI preoperatively, take no account of tumor size or diameter. The AUCs of aforementioned scoring system were 0.872 and 0.856 among training cohort as well validation cohort, respectively. Lei et al. (32) built a nomogram scoring model with risk factors such as tumor diameter, number, vessel condition, serum AFP content, platelet level, HBV-DNA loading in addition with representative dynamical magnetic resonance imaging features for predicting MVI occurrence among patients suffered from HBV-related HCC under the Milan criteria, which shows satisfactory predictive efficiency whose AUC was 0.81 among training cohort also 0.80 among validation cohort. Similarly, Renzulli et al. (9) applied integration of three imaging features of predictive significance, gaining an AUC of 0.90. In terms of clinicopathological risk factors among our study, AFP, neutrophilic granulocyte, and preoperative hemoglobin content were of greater importance than the Rad-score in the regression model. When applied in the validation cohort, the discernibility ability of proposed model outperformed three conventional method mentioned in this article. For calibration curve analysis, the result in both cohorts are satisfying, furthermore, the result of decision curve analysis showed this proposed model possessed clinical application potential. The result of pilot study demonstrated that the accuracy of this model is preferably among study data set (whose AUC was 0.844, 95% confidential interval was 0.628–1.000).

### Limitations

There are certainly some underlying limitations to this study. First, the analysis results suffer from inherent selection biases without a large enough number of included patients. Second, due to the study cohort selection from a single center, which could not offer sufficient diversity of characteristics to be representative of the general population, the lack of external validation from other centers was a shortcoming of this study. Moreover, heterogeneity of CT images will give rise to bias during the procedures as image features extraction and segmentation for region of interest. In the follow-up study, a larger study cohort and external validation will test and improve the efficacy and feasibility of this model for patient management and clinical decision-making for MVI prediction in HCC. Therefore, unified standards in addition with elaborated and rigorous assessment for quality of study images should be established in order to acquire more reliable study data.

## Conclusions

We have built and evaluated a novel radiomics feature-based nomogram to predict the incidence of MVI among patients suffered from HCC. the purposed nomogram has provided one novel method for risk identification of MVI incidence and of radiomics features as a supplementary resource to clinicopathological data and conventional CT images that could lower the medical costs and improve the availability of clinical decision-making and treatment scheme guidance.

## Data Availability Statement 

The raw data supporting the conclusions of this article will be made available by the authors, without undue reservation.

## Ethics Statement

The studies involving human participants were reviewed and approved by The Ethics Review Board of Zhujiang Hospital, Southern Medical University. The patients/participants provided their written informed consent to participate in this study.

## Author Contributions

CF and FJ conceived of the presented idea. PZ collected the data. MH, XM, BH, and FJ analyzed the data. MH drafted the manuscript. All authors reviewed the manuscript and FJ made corrections to the manuscript. All authors contributed to the article and approved the submitted version.

## Funding

This work was supported by grants from the National Key R&D Program, China (Nos. 2019YFC0118100 and 2016YFC0106500), the Major Instrument Project of National Natural Science Fund of China (No. 81627805), the Shenzhen Key Basic Science Program (JCYJ20170413162213765 and JCYJ20180507182437217), the NSFC-Shenzhen Union Program (U1613221), the Youth Program of Natural Science Foundation of Anhui Province (2008085QH418), the Key-Area Research and Development Program of Guangdong Province (2020B010165004), and the National Key Research and Development Program (2017YFC0110903).

## Conflict of Interest

The authors declare that the research was conducted in the absence of any commercial or financial relationships that could be construed as a potential conflict of interest.
